# Predictive Factors for Small Head Circumference Among Pregnant Women with Third-Trimester Anemia

**DOI:** 10.3390/ijerph23080964

**Published:** 2026-07-26

**Authors:** Rachadaporn Jantasuwan, Suda Jaihow, Sumoltip Sontimuang, Kanyapak Pluemjai, Hasni Embong, Salwismawati Badrin

**Affiliations:** 1 Division of Community Nursing, School of Nursing, The Excellence Center of Community Health Promotion, Walailak University, Nakhon Si Thammarat 80161, Thailand; rachadaporn.jn@wu.ac.th; 2 Division of Maternal-Newborn and Midwifery Nursing, School of Nursing, The Excellence Center of Community Health Promotion, Walailak University, Nakhon Si Thammarat 80161, Thailand; kanyapak.pl@wu.ac.th; 3 Division of Pediatric Nursing, School of Nursing, The Excellence Center of Community Health Promotion, Walailak University, Nakhon Si Thammarat 80161, Thailand; poysontimuang@gmail.com; 4 Nursing Program, School of Health Sciences, Universiti Sains Malaysia, Health Campus, Kota Bharu 16050, Malaysia; ehasni@usm.my (H.E.); salwis@usm.my (S.B.)

**Keywords:** maternal anemia, gravidity, gestational weight gain, pre-pregnancy body mass index

## Abstract

**Highlights:**

**Public health relevance—how does this work relate to a public health issue?**

- This study addresses an important maternal and child health issue by examining predictors of small head circumference (HC) among infants born to women with third-trimester anemia, a condition that may adversely affect fetal growth and neurodevelopment.
- Small HC at birth may reflect impaired fetal brain growth and is associated with adverse developmental outcomes. Identifying maternal risk factors may facilitate early detection and prevention of unfavorable neonatal outcomes.

**Public health significance—why is this work of significance to public health?**

- The findings provide evidence on modifiable maternal factors, including gravidity, pre-pregnancy body mass index (BMI), and gestational weight gain (GWG), that influence neonatal HC among women with third-trimester anemia.
- Understanding these predictors may support the development of targeted antenatal interventions aimed at optimizing maternal nutritional status and improving fetal growth outcomes.

**Public health implications—what are the key implications or messages for practitioners, policy makers and/or researchers in public health?**

- Healthcare providers should closely monitor GWG among pregnant women with anemia, particularly primigravida women and those with suboptimal pre-pregnancy BMI, to reduce the risk of small HC at birth.
- Policymakers and public health practitioners should strengthen maternal nutrition programs, anemia prevention strategies, and antenatal screening services to promote optimal fetal growth and neurodevelopment.
- Future research should investigate effective interventions to optimize pre-pregnancy BMI and achieve appropriate GWG, with particular emphasis on primigravida women and those at high risk of anemia to improve HC at birth.

**Abstract:**

Maternal anemia during pregnancy may adversely affect fetal growth, including neonatal head circumference (HC). This retrospective cohort study aimed to identify predictors of small HC among infants born to women with third-trimester anemia. Secondary data were obtained from the HosXP electronic medical database and delivery records of pregnant women who delivered between October 2020 and September 2023. A total of 934 women met the eligibility criteria and were included in the analysis. Maternal characteristics were summarized using descriptive statistics. Associations between maternal factors and neonatal HC were examined using the Chi-square test, Fisher’s exact test or Mann–Whitney U test, and independent predictors were identified using binary logistic regression analysis. The mean neonatal HC was 32.76 ± 1.50 cm, and 43.90% of infants were classified as having a small HC. Gravidity, pre-pregnancy body mass index (BMI), and gestational weight gain (GWG) were significantly associated with neonatal HC. Multivariable analysis revealed that women with two pregnancies (aOR = 0.51, 95% CI: 0.352–0.734) and those with three or more pregnancies (aOR = 0.54, 95% CI: 0.373–0.773) had lower odds of delivering infants with small HC than primigravida women. Pre-pregnancy overweight or obesity was associated with lower odds of small HC (aOR = 0.59, 95% CI: 0.426–0.805), whereas inadequate GWG increased the likelihood of this outcome (aOR = 1.38, 95% CI: 1.010–1.894). Maternal gravidity, pre-pregnancy BMI, and GWG may influence neonatal HC. These findings may support nurses in identifying women at risk, monitoring GWG, and providing targeted nutritional counseling during antenatal care.

## 1. Introduction

Anemia remains one of the most common medical complications during pregnancy and poses substantial risks to both maternal and fetal health. Despite ongoing efforts to improve maternal nutrition and antenatal care, anemia continues to affect a considerable proportion of pregnant women worldwide. Recent evidence has estimated the prevalence of anemia during pregnancy to range from 36.80% to 54.00%, highlighting its persistent public health burden [1,2]. Mild anemia accounts for the majority of cases, with a pooled prevalence of 70.80% (95% CI: 58.10–810). The condition is most frequently observed during the third trimester, when the prevalence reaches 48.80% (95% CI: 38.70–58.90), reflecting the increased physiological demands of late pregnancy. In Asia, a large-scale study conducted in China reported that 30.70% of pregnant women experienced anemia [3].

Anemia during pregnancy is a multifactorial condition resulting from both nutritional and physiological factors. Nutritional deficiencies, particularly iron, folate, and vitamin B12 deficiencies, are among the leading causes. Other contributing factors include maternal undernutrition, low pre-pregnancy body mass index (BMI), parasitic infections, hemoglobin disorders, adolescent pregnancy, inadequate antenatal care, and multiple gestations [4]. In addition to these pathological causes, physiological changes during pregnancy may contribute to reduced hemoglobin levels. Maternal plasma volume expands by approximately 40–50% above pre-pregnancy levels, beginning in early gestation, reaching its peak around 32 weeks, and subsequently plateauing. Because this increase exceeds the approximately 30% rise in red blood cell mass, a relative hemodilution occurs, resulting in lower hemoglobin concentrations. This normal physiological adaptation, known as the physiological anemia of pregnancy, is especially evident during the third trimester of pregnancy [3,5].

Maternal anemia during pregnancy has been linked to a range of adverse fetal outcomes, including preterm birth, low birth weight [6], and fetal growth restriction [4]. Emerging evidence also suggests that maternal anemia may negatively affect neonatal head circumference (HC), an important indicator of fetal brain growth and development [7]. Among full-term newborns, the normal HC typically ranges from 33 to 37 cm [8]. Maternal anemia may compromise fetal oxygenation and nutrient supply, both of which are essential for normal fetal growth and brain development. Inadequate delivery of these critical substrates during pregnancy may restrict cranial growth and contribute to a reduced HC at birth. Previous evidence has shown that pregnant women with anemia were 2.61 times more likely to deliver infants with low HC than women without anemia (AOR = 2.61, 95% CI: 1.18–5.78) [9]. Consistent with these findings, neonates born to mothers with anemia had significantly smaller mean HC measurements than those born to mothers without anemia (31.56 ± 1.05 cm vs. 32.26 ± 1.44 cm, *p* = 0.01) [6]. Similar results were reported in another study, which demonstrated that newborns of anemic mothers had significantly lower HC values compared with newborns of non-anemic mothers (33.40 ± 1.20 cm vs. 34.70 ± 1.30, *p* < 0.001) [10]. These findings further support the association between maternal anemia and impaired fetal cranial growth.

A small HC in a newborn can result from several factors. Studies have shown that maternal anemia before conception was associated with HC. The greatest benefits of preconception nutritional supplementation were observed among women with hemoglobin concentrations of 90–99 g/L, resulting in significant improvements in neonatal HC at birth [11]. Neonatal HC showed a declining trend with increasing severity of maternal anemia. The mean HC was 33.70 ± 2.10 cm among mothers with normal hemoglobin levels, 33.70 ± 1.60 cm among those with mild anemia, 32.90 ± 2.40 cm among those with moderate anemia, and 31.40 ± 3.80 cm among those with severe anemia, indicating progressively reduced fetal cranial growth as maternal anemia became more severe [12]. Higher gravidity has been reported to correlate significantly with larger neonatal HC measurements (r = 0.311, *p* = 0.028), suggesting that women with previous pregnancies may have more favorable conditions for fetal cranial growth than primigravida women [13]. Similarly, evidence from a large population-based study indicated that primiparity was associated with a significantly increased risk of mild microcephaly, with first-time mothers having 1.70 times higher odds of delivering infants with small HC compared with multiparous women (OR = 1.70, 95% CI: 1.50–2.00) [14]. Moreover, maternal age is an important determinant of fetal growth and neonatal anthropometric outcomes, including HC. Previous studies have shown that infants born to adolescent mothers and women of advanced maternal age are more likely to have smaller HC measurements than those born to mothers of optimal reproductive age [15].

Furthermore, a study conducted in Iran found that infants born to underweight mothers had significantly smaller HC measurements than those born to obese mothers. A pre-pregnancy BMI < 18.50 kg/m^2^ was associated with a 1.06 cm reduction in neonatal HC compared with a BMI ≥ 30.00 kg/m^2^ (*p* = 0.002) [16]. In addition, severely inadequate gestational weight gain (GWG) was associated with a 57% increased risk of microcephaly (aRR = 1.57, 95% CI: 1.31–1.88) [17]. Moreover, previous evidence has demonstrated that inadequate GWG may adversely affect fetal cranial growth. In particular, severely inadequate GWG was associated with a 57% increased risk of microcephaly (aRR = 1.57, 95% CI: 1.31–1.88), suggesting that suboptimal maternal nutritional status during pregnancy may contribute to reduced head growth and smaller HC at birth [17].

Although maternal anemia has been associated with impaired fetal growth and reduced neonatal HC, evidence regarding predictors of small HC among women with third-trimester anemia remains scarce. A better understanding of these predictors may facilitate the identification of high-risk pregnancies and support the development of effective preventive strategies. Therefore, this study aimed to identify factors predicting small HC among infants born to women with third-trimester anemia. The findings may provide evidence to guide antenatal care interventions and improve fetal growth and birth outcomes.

## 2. Materials and Methods

### 2.1. Research Design and Participants

A retrospective cohort study was conducted to investigate factors predicting small HC among infants born to women with third-trimester anemia. Secondary data were obtained from a previously approved research project entitled “Factors Related to Anemia and Birth Outcomes of Women Attending Antenatal Care at Thasala Hospital, Nakhon Si Thammarat Province,” following authorization from the principal investigator. The dataset was derived from the HosXP electronic medical database and delivery records of pregnant women who delivered between 1 October 2020 and 30 September 2023. A total of 999 women with documented third-trimester anemia were initially identified for screening and eligibility assessment.

### 2.2. Inclusion and Exclusion Criteria

Participants were eligible for inclusion if they met the following criteria: (1) were Thai nationals and (2) had anemia during the third trimester of pregnancy, defined as a hematocrit level below 33% or a hemoglobin concentration below 11 g/dL. Participants were excluded if (1) neonatal HC data were unavailable or missing, or (2) the infant was born preterm. Following the application of the inclusion and exclusion criteria, a total of 934 pregnant women were retained for the final analysis.

### 2.3. Research Instruments

Data were collected using a structured data extraction form developed by the researchers. Variables were selected based on a review of the medical literature on predictors of small neonatal HC. The section 1 collected participants’ demographic characteristics, including maternal age during the index pregnancy, educational attainment, marital status, and religion. The section 2 recorded maternal and pregnancy-related factors potentially associated with neonatal HC, including gravidity, gestational age at the first antenatal care (ANC) visit, number of quality ANC visits, pre-pregnancy BMI, GWG, and hemoglobin and hematocrit levels. The section 3 documented neonatal outcomes, including neonatal HC at birth.

Neonatal HC was classified according to standard deviation (SD)-based criteria, consistent with the World Health Organization recommendation for defining normal growth limits. Measurements within ±2 SD of the mean (33–37 cm at birth) were considered optimal HC. Values below 33 cm (>2 SD below the mean) were classified as below-optimal HC, whereas values above 37 cm (>2 SD above the mean) were classified as above-optimal HC. For the purpose of this study, infants with a HC < 33 cm were categorized as having small HC [8].

### 2.4. Data Analysis

Participant characteristics were summarized using descriptive statistics and reported as frequencies and percentages. Associations between maternal characteristics and small HC were initially examined using the Chi-square test or Fisher’s exact test or Mann–Whitney U test, as appropriate. Variables demonstrating a significance level of *p* < 0.20 in the analysis, together with relevant demographic characteristics, were subsequently entered into a binary logistic regression model to identify independent predictors of small HC. The significance of the logistic regression model was assessed using the Omnibus test of model coefficients. Model fit was evaluated using the Hosmer–Lemeshow goodness-of-fit test, and model performance was summarized using the Nagelkerke R^2^ statistic. The results were expressed as adjusted odds ratios (aORs) with corresponding 95% confidence intervals (CIs). Statistical significance was established at *p* < 0.05.

The adequacy of the sample size for logistic regression analysis was evaluated based on the events-per-variable (EPV) principle. Previous simulation studies have suggested that a minimum of 10 outcome events per predictor variable is required to produce stable and reliable parameter estimates in logistic regression models [18]. Given the number of small HC events observed in the present study, the sample size was considered sufficient to support the validity and robustness of the multivariable regression analysis.

### 2.5. Ethical Consideration

The study received exemption review approval from the Committee on Human Rights Related to Research Involving Human Subjects, Walailak University, Thailand (Approval No. WUEC-26-207-01). The ethical approval was valid from 12 May 2026 to 11 May 2027.

## 3. Results

### 3.1. Participant Characteristics

A total of 934 pregnant women with third-trimester anemia were included in the analysis. The majority of participants were aged 20–34 years (74.84%), followed by those of advanced maternal age (15.95%). Most participants had completed secondary school or an equivalent level of education (60.28%), while 19.38% held a bachelor’s degree or higher. Nearly all participants were married (99.68%), and more than two-thirds identified Buddhism as their religion (71.73%). Regarding neonatal outcomes, the mean neonatal HC was 32.76 ± 1.50 cm. Overall, 43.90% of infants were classified as having a small HC. The prevalence of small HC was highest among adolescent mothers aged 19 years or younger, with 57.00% of infants in this age group exhibiting small HC. Detailed participant characteristics stratified by HC status are presented in Table 1.

### 3.2. Factors Associated with HC

Several maternal factors were significantly associated with neonatal HC. Gravidity, pre-pregnancy BMI, and GWG showed statistically significant associations with HC (*p* < 0.001, *p* < 0.001, and *p* < 0.01, respectively). Among women with moderate anemia, the median third-trimester hemoglobin level was significantly lower in the small HC group than in the optimal HC group (9.42 [IQR 0.72] vs. 9.60 [IQR 0.63] g/dL; *p* = 0.013). The highest proportion of small HC was observed among primigravida women (56.64%). With respect to pre-pregnancy BMI, the prevalence of small HC was greatest among women who were underweight prior to pregnancy (52.55%). Similarly, infants born to mothers with below-recommended GWG exhibited the highest prevalence of small HC (49.55%). Detailed results are presented in Table 2.

### 3.3. Predictors of Small HC

Multivariable binary logistic regression analysis identified gravidity, pre-pregnancy BMI, and GWG as significant predictors of small HC. Compared with primigravida women, those with two pregnancies and those with three or more pregnancies had significantly lower odds of delivering infants with small HC (aOR = 0.51, 95% CI: 0.352–0.734, *p* < 0.001; and aOR = 0.54, 95% CI: 0.373–0.773, *p* = 0.001, respectively). In addition, women who were overweight or obese before pregnancy had significantly lower odds of having infants with small HC compared with those with a normal pre-pregnancy BMI (aOR = 0.59, 95% CI: 0.426–0.805, *p* = 0.001). Conversely, inadequate GWG was associated with a significantly increased likelihood of small HC compared with optimal GWG (aOR = 1.38, 95% CI: 1.010–1.904, *p* = 0.043). Detailed results of the multivariable analysis are presented in Table 3.

Overall, small HC was a common neonatal outcome among women with third-trimester anemia. Maternal gravidity, pre-pregnancy BMI, and GWG were identified as significant determinants of neonatal HC. Specifically, multiparity and pre-pregnancy overweight/obesity were associated with reduced odds of small HC, whereas inadequate GWG increased the likelihood of this outcome. These variables were retained as independent predictors in the adjusted logistic regression model.

## 4. Discussion

The highest prevalence of small HC was observed among adolescent mothers aged 19 years or younger, with 57.00% of infants in this age group classified as having small HC. This finding is consistent with previous research demonstrating that neonates born to mothers aged ≤19 years had significantly smaller HC measurements than those born to mothers aged 20 years and older [19]. A possible explanation is that adolescent mothers are still undergoing physical growth and development, resulting in competition for nutrients between the mother and the fetus, particularly during the third trimester when fetal brain growth accelerates. Consequently, the availability of nutrients required for optimal fetal growth and cranial development may be reduced, leading to smaller neonatal HC [15].

Gravidity, pre-pregnancy BMI, and GWG were significantly associated with neonatal HC and remained significant predictors after adjustment for potential confounders. Specifically, multiparous women and those who were overweight or obese before pregnancy were less likely to deliver infants with small HC, whereas inadequate GWG led to the likelihood of this outcome.

The present study found that women with two pregnancies and those with three or more pregnancies were significantly less likely to deliver infants with small HC than primigravida women. This finding is inconsistent with a previous study that reported a significant association between parity and low neonatal HC among anemic mothers [20]. A possible explanation for the protective effect observed among multiparous women is related to physiological adaptations of the uteroplacental circulation. During the first pregnancy, extravillous trophoblast invasion and remodeling of the uterine spiral arteries occur for the first time. Inadequate remodeling may result in persistent high-resistance spiral arteries, reduced uteroplacental perfusion, placental malperfusion, and subsequent fetal growth restriction, all of which may adversely affect fetal brain growth and HC [21,22,23]. Following a previous pregnancy, structural and functional adaptations of the uterine vasculature may persist, a phenomenon often referred to as “vascular memory.” These adaptations facilitate more efficient uteroplacental blood flow and lower vascular resistance in subsequent pregnancies, thereby enhancing the delivery of oxygen and nutrients to the developing fetus [24,25].

Another possible explanation is that women with previous pregnancy experience may be more likely to seek antenatal care earlier and adhere to recommended nutritional interventions, including iron supplementation. Early and adequate iron supplementation has been shown to improve fetal growth parameters. Previous evidence demonstrated that iron supplementation during the first and second trimesters, as well as higher cumulative iron intake throughout pregnancy, was positively associated with fetal growth, including increased biparietal diameter z-scores, an important indicator of fetal cranial growth [26]. Collectively, these findings suggest that both physiological adaptation and improved maternal health behaviors in subsequent pregnancies may contribute to the lower risk of small HC observed among multiparous women.

The present study found that mothers who were overweight or obese before pregnancy had significantly lower odds of delivering infants with small HC than mothers with a normal pre-pregnancy BMI. This finding is consistent with previous research from Iran, which reported that infants born to underweight mothers had significantly smaller HC measurements than those born to obese mothers [16]. Similarly, a previous Thai study involving pregnant women with and without anemia found that pre-pregnancy overweight or obesity was associated with lower odds of small neonatal HC compared with normal pre-pregnancy BMI (aOR = 0.71, 95% CI: 0.62–0.82), supporting the findings of the present study [27]. A possible explanation for this finding can be drawn from the Maternal Nutritional Buffering Model, which proposes that women with higher pre-pregnancy BMI possess greater energy and nutrient reserves that can support fetal growth throughout pregnancy. These reserves provide a continuous source of energy, protein, and essential micronutrients required for placental function and fetal development. Consequently, mothers with higher BMI may be better able to maintain nutrient delivery to the fetus, even under conditions of increased physiological demand. Furthermore, maternal metabolic adaptations and stored nutrient reserves may help stabilize nutrient transfer across the placenta despite short-term fluctuations in dietary intake during pregnancy. Among women with anemia, these compensatory mechanisms may partially mitigate the adverse effects of reduced oxygen-carrying capacity and support fetal brain growth, thereby lowering the risk of small HC at birth [28].

The present study found that inadequate GWG was associated with a significantly increased risk of small HC among infants born to women with third-trimester anemia. This finding may be explained by insufficient maternal energy and nutrient intake during pregnancy, particularly iron, protein, and other micronutrients essential for fetal growth and brain development. In women with anemia, inadequate GWG may further compromise maternal nutritional reserves, thereby limiting the availability of oxygen and nutrients required for optimal fetal development. Suboptimal maternal nutritional status may impair placental development and function, leading to uteroplacental malperfusion and placental insufficiency. Reduced placental blood flow and oxygen delivery can induce cellular stress within the placenta, resulting in decreased protein synthesis, impaired cell proliferation, placental infarction, and excessive fibrin deposition in severe cases. These pathological changes reduce the volume of chorionic villi and the surface area available for maternal–fetal nutrient exchange, thereby restricting the transfer of oxygen and essential nutrients to the fetus. When such insults occur during critical periods of fetal brain development, they may disrupt neurogenesis and synaptogenesis, ultimately impairing brain growth and increasing the risk of reduced HC at birth [22]. This finding is consistent with previous evidence demonstrating that inadequate GWG adversely affects fetal cranial growth. Women with moderately and severely inadequate GWG had a higher risk of delivering infants with microcephaly, respectively, compared with women who achieved adequate GWG (RR = 1.26, 95% CI: 1.12–1.41; and RR = 1.57, 95% CI: 1.31–1.88, respectively) [17]. Additionally, although hemoglobin levels differed significantly between the two neonatal HC groups in the univariate analysis among women with moderate anemia, hemoglobin was not an independent predictor of small HC after adjustment for maternal characteristics in the multivariable logistic regression model (aOR = 1.017, *p* = 0.878). This finding suggests that maternal gravidity, pre-pregnancy BMI, and GWG had stronger independent associations with neonatal HC than hemoglobin level in this study population.

## 5. Strengths and Limitations

This study has several strengths. It included a large sample of women with third-trimester anemia and evaluated multiple maternal predictors of neonatal HC. However, several limitations should be acknowledged. First, this was a secondary analysis of routinely collected medical records; therefore, the available variables were restricted to those recorded in the hospital database. Information on maternal smoking, alcohol consumption, nutritional supplementation, adherence to iron therapy, placental abnormalities, and other potential confounding factors was unavailable. Consequently, residual confounding cannot be excluded. Second, because this was a retrospective cohort study based on a single hospital, the findings may not be generalizable to other populations or healthcare settings.

## 6. Conclusions

This study identified maternal gravidity, pre-pregnancy BMI, and GWG as significant predictors of small HC among infants born to women with third-trimester anemia. Multiparous women and those who were overweight or obese before pregnancy had a lower likelihood of delivering infants with small HC, whereas inadequate GWG was associated with an increased risk of this outcome. These findings underscore the importance of maternal nutritional status and appropriate GWG in supporting fetal head growth. Nurses should perform early risk assessment, monitor GWG, and provide individualized nutritional counseling, particularly for primigravida women and those with underweight pre-pregnancy BMI, to support optimal HC. Moreover, future prospective studies should:

(1) Include additional clinical confounders, such as maternal smoking, alcohol use, nutritional supplementation, iron therapy adherence, maternal chronic diseases, placental abnormalities, and other factors associated with neonatal head circumference (e.g., fetal alcohol syndrome).
(2) Standardize neonatal HC measurements by recording the mean of three measurements at the maximum occipitofrontal circumference.
(3) Conduct longitudinal follow-up of head circumference from birth to 3 years of age to evaluate postnatal growth trajectories and neurodevelopmental outcomes.

## Figures and Tables

**Table 1:** Characteristics of participants according to HC category (*n* = 934).

General Characteristic	Overall *n* (%)	HC (cm) ($\bar{x}$ ± SD = 32.76 ± 1.50, Min = 29, Max = 39)			
		Small (<33) *n* (%)	Optimal/Above (≥33) *n* (%)	χ^2^/F	*p*-Value
	934 (100.00)	410 (43.90)	524 (56.10)		
Age (Year)				6.65	0.036 *
≤19	86 (9.21)	49 (57.00)	37 (43.00)		
20–34	699 (74.84)	299 (42.78)	400 (57.22)		
≥35	149 (15.95)	62 (41.61)	87 (58.39)		
Educational Level				2.01	0.571
Below Secondary School	134 (14.35)	65 (48.51)	69 (51.49)		
Secondary School or Equivalent	563 (60.28)	248 (44.05)	315 (55.95)		
Diploma/Associate Degree	56 (5.99)	23 (41.07)	33 (58.93)		
Bachelor’s Degree or Higher	181 (19.38)	74 (40.88)	107 (59.12)		
Marital Status				2.39 ^F^	0.331
Married	931 (99.68)	409 (43.93)	522 (56.07)		
Divorced	1 (0.11)	1 (100.00)	0 (0.00)		
Widowed	2 (0.21)	0 (0.00)	2 (100.00)		
Religion				0.00	1.000
Buddhism	670 (71.73)	294 (43.88)	376 (56.12)		
Islam	264 (28.27)	116 (43.94)	148 (56.06)		

χ^2^ = chi-square test; ^F^ = Fisher’s exact test; * *p* < 0.05.

**Table 2:** Factors associated with HC among pregnant women with third-trimester anemia (*n* = 934).

Associated Factors	Overall *n* (%)	HC (cm)			
		Small (<33) *n* (%)	Optimal/Above (≥33) *n* (%)	χ^2^/F/U	*p*-Value
	934 (100.00)	410 (43.90)	524 (56.10)		
Gravidity				23.26	0.000 ***
Primigravida	256 (27.41)	145 (56.64)	111 (43.36)		
Gravida 2	290 (31.05)	113 (38.97)	177 (61.03)		
Gravida ≥ 3	388 (41.54)	152 (39.18)	236 (60.82)		
GA at first ANC				1.02	0.320
≤12 wks	518 (55.46)	235 (45.37)	283 (54.63)		
>12 wks	416 (44.54)	175 (42.07)	241 (57.93)		
Number of quality ANC				1.28	0.268
<8 times	170 (18.20)	68 (40.00)	102 (60.00)		
≥8 times	764 (81.80)	342 (44.76)	422 (55.24)		
Hemoglobin (Hb)					
Moderate anemia	Median (IQR) = 9.53 (0.70)	Median (IQR) = 9.42 (0.72)	Median (IQR) = 9.60 (0.63)	4694.00 ^U^	0.013 *
Mild anemia	Median (IQR) = 10.60 (0.40)	Median (IQR) = 10.43 (0.67)	Median (IQR) = 10.33 (0.67)	61213.50	0.485
Severity of anemia				0.41	0.534
Moderate (Hb 7–9.99 g/dL)	219 (23.45)	92 (42.01)	127 (57.99)		
Mild (Hb 10–10.99 g/dL)	715 (76.55)	318 (44.48)	397 (55.52)		
Underlying diseases					
No	841 (90.04)	363 (43.16)	478 (56.84)	5.056 ^F^	0.290
Thalassemia	19 (2.03)	8 (42.11)	11 (57.89)		
DM/GDM	54 (5.78)	29 (53.70)	25 (46.30)		
HT/PIH	15 (1.61)	6 (40.00)	9 (60.00)		
Others	5 (0.54)	4 (80.00)	1 (20.00)		
Pre-pregnancy BMI				25.47	0.000 ***
Underweight	137 (14.67)	72 (52.55)	65 (47.45)		
Normal weight	513 (54.93)	248 (48.34)	265 (51.66)		
Overweight/Obesity	284 (30.40)	90 (31.69)	194 (68.31)		
GWG				12.61	0.002 **
Below	448 (47.97)	222 (49.55)	226 (50.45)		
Optimal	278 (29.76)	114 (41.01)	164 (58.99)		
Above	208 (22.27)	74 (35.58)	134 (64.42)		

χ^2^ = chi-square test; ^F^ = Fisher’s exact test; ^U^ = Mann–Whitney U test, * *p* < 0.05; ** *p* < 0.01; *** *p* < 0.001.

**Table 3:** Multivariable Binary Logistic Regression Analysis of Factors Associated with Small HC among Pregnant Women with Third-Trimester Anemia (*n* = 934).

Predictive Factors	Overall	HC (cm)			
		Small (<33)	Optimal/Above (≥33)	aOR (95% CI)	*p*-Value
	*n* (%)	*n* (%)	*n* (%)		
	934 (100.00)	410 (43.90)	524 (56.10)		
Age (Year)					0.773
20–34 ^Ref^	699 (74.84)	299 (42.78)	400 (57.22)		
≤19	86 (9.21)	49 (57.00)	37 (43.00)	1.19 (0.727–1.953)	0.487
≥35	149 (15.95)	62 (41.61)	87 (58.39)	1.04 (0.713–1.528)	0.827
Gravidity					0.001 **
Primigravida ^Ref.^	256 (27.41)	145 (56.64)	111 (43.40)		
Gravida 2	290 (31.05)	113 (39.97)	177 (61.03)	0.51 (0.352–0.734)	0.000 ***
Gravida ≥ 3	388 (41.51)	152 (39.18)	236 (60.82)	0.54 (0.373–0.773)	0.001 **
BMI					0.002
Normal weight ^Ref.^	513 (54.93)	248 (48.34)	265 (51.66)		
Underweight	137 (14.67)	72 (52.55)	65 (47.45)	1.09 (0.742–1.612)	0.650
Overweight/Obesity	284 (30.41)	90 (31.69)	194 (68.31)	0.59 (0.426–0.805)	0.001 **
GWG					0.022 *
Optimal ^Ref.^	278 (29.76)	114 (41.01)	164 (58.99)		
Below	448(47.97)	222 (49.55)	226 (50.45)	1.38 (1.010–1.904)	0.043 *
Above	208 (22.27)	74 (35.58)	134 (64.42)	0.87 (0.591–1.277)	0.474
Hemoglobin (g/dL)	($\bar{x}$ ± SD = 10.24 ± 0.62, Min = 7.30, Max = 10.97)	($\bar{x}$ ± SD = 10.24 ± 0.66, Min = 7.30, Max = 10.97)	($\bar{x}$ ± SD = 10.24 ± 0.58, Min = 7.33, Max = 10.97)	1.017 (0.817–1.267)	0.878

Model fit: Omnibus test, *p* < 0.001; Nagelkerke R^2^ = 0.073; Hosmer–Lemeshow goodness-of-fit test, *p* = 0.887. aOR = Adjusted Odds Ratio; ^Ref^ = Reference; * *p* < 0.05; ** *p* < 0.01; *** *p* < 0.001.

## Data Availability

The datasets analyzed during the current study are not publicly available because they contain sensitive information and are subject to ethical and institutional restrictions. Data may be made available from the corresponding author, Rachadaporn Jantasuwan, upon reasonable request and with appropriate approval from the relevant authorities.
